# Design of a miniature sensing module for pressure mapping in functionality hydrogel using programmable system-on-chip

**DOI:** 10.1016/j.ohx.2026.e00762

**Published:** 2026-03-20

**Authors:** Zhuwen Xu, Yin Zhang, Zeyu Liu, Xingqi Lu, Runhuai Yang, Shengzhao Zhang

**Affiliations:** School of Biomedical Engineering, Anhui Medical University, Hefei 230032, China

**Keywords:** Multi-channel acquisition system, Miniaturization, Square ring array, Programmable system-on-chip

## Abstract

Flexible tactile sensors show great promise in mimicking human skin to achieve tactile perception. However, their practical implementation still faces key technical challenges, including device miniaturization, high-sensitivity detection, and suppression of signal crosstalk. In this work, we propose a micro multi-channel pressure collection and monitoring system for functional hydrogels, which integrates a sensor electrode array with a downsized multi-channel acquisition module for efficient pressure sensing. The hydrogel material was combined with the electrode array to form the sensing units. To address the influence of different array structures on the integrity of the hydrogel, an independent array design is adopted to reduce interference from redundant conductive pathways. The array is downscaled to a 4 × 4 structure with a size of 1.8 cm × 1.8 cm. The system employs a conversion circuit to provide alternating current excitation, which helps mitigate electrochemical corrosion caused by redox reactions between the hydrogel and metal electrodes. The system consists of a programmable system-on-chip (PSoC) and a multiplexer, enabling multi-pixel tactile array measurement while minimizing the use of additional components. The acquisition circuit is compact, with dimensions of only 2.3 cm × 3.0 cm. The array characteristics were validated by testing individual pixel elements, and experiments such as pressing concave and convex letter patterns as well as vascular structure detection were conducted, with vascular structures successfully displayed on the interface. This system enables real-time tactile measurement through touch, demonstrating significant potential in applications such as human–machine interaction and electronic skin.

## Graphical abstract

**Figure:**
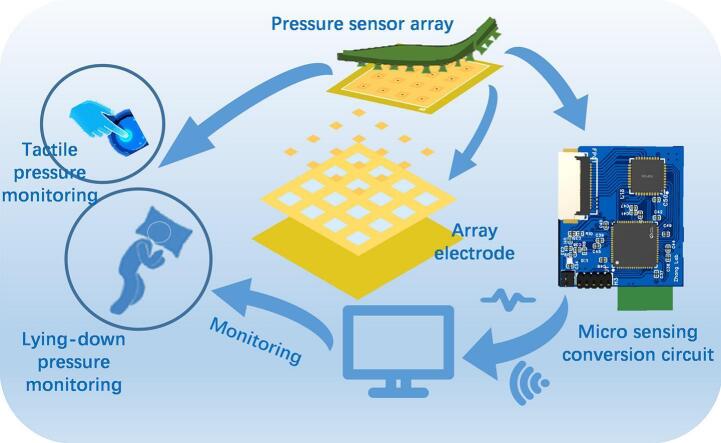

**Table:** 

**Specifications table**	
Hardware name	Wireless micro multi-channel pressure monitoring system
Subject area	Engineering and material science
Hardware type	Field measurements and sensors
Closest commercial analog	Data Acquisition Card(DAQ)
Open source license	CC BY 4.0
Cost of hardware	172.86
Source file repository	https://data.mendeley.com/datasets/6n9t58g2v3/1

## 1 Hardware in context

Tactile sensors, as an essential component of flexible electronics, have been widely applied in robotics, health monitoring, and human–machine interaction [1], [2], [3], [4], among which pressure-sensing arrays serve as the core hardware for achieving spatially distributed measurements. Existing pressure-sensing mechanisms include piezoelectric effect [5], [6], [7], triboelectric effect [8], [9], [10], piezoresistive effect [11], [12], [13], and capacitive effect [14], [15], [16]. Among these, the piezoresistive effect has been extensively employed in array sensors due to its simple circuitry and strong anti-interference capability [5]. However, sensors based on traditional rigid materials are difficult to adapt to complex surfaces, which limits their application in flexible systems. In recent years, flexible hydrogels, owing to their excellent conformability and biocompatibility, have been introduced into sensing arrays, providing a new material foundation for high-sensitivity flexible sensing. In particular, functional hydrogels exhibit unique properties such as electrical conductivity and antibacterial activity, enabling advanced features including self-healing and multimodal perception [17], [18]. These advantages not only enhance the stability and sensitivity of sensors under complex conditions but also broaden their application potential in electronic skin, soft robotics, implantable devices, and real-time health monitoring [19], [20], [21]. Therefore, integrating functional hydrogels with a programmable system-on-chip to construct a miniaturized pressure mapping sensor module is of great research significance and has broad application prospects.

In tactile sensing applications, the key challenges extend beyond the choice of sensing materials to the holistic optimization of array structures and signal acquisition circuits. Wearable sensors have attracted considerable attention as a promising technology for continuous, real-time monitoring of human health and performance, where miniaturized designs offer distinct advantages in portability and comfort [22], [23], [24]. However, most reported tactile sensing systems remain bulky, limiting their conformal integration with complex surfaces and their ability to perform high-resolution measurements [25], [26], [27], [28]. This underscores the urgent need for miniaturization in both sensor arrays and acquisition circuits.

Spatially resolved measurements are typically achieved through array electrical designs, yet a persistent challenge lies in realizing high-precision, low-crosstalk distributed force sensing. Many existing hydrogel arrays rely on discrete-material array structures to achieve spatial resolution [4], [29], [30]. While effective, these designs are generally large in size and constrained in resolution, restricting their applicability in miniaturized and high-precision scenarios. By contrast, continuous-material structures can reduce device size and simplify fabrication, but they often suffer from pronounced mechanical and electrical crosstalk [31], [32], [33]. Mechanical crosstalk arises when force applied to one pixel affects neighboring pixels, leading to measurement errors. This issue is commonly addressed by optimizing pixel density to enhance spatial independence and reduce interference [34], [35], [36]. Electrical crosstalk, in contrast, stems from unintended conductive pathways or current leakage. Conventional row–column scanning structures exacerbate this problem by introducing external circuit effects and internal current pathways, resulting in inaccurate measurements. Strategies such as integrating isolated sensing elements within a common electrode layer have been proposed to mitigate crosstalk [37], [38]. Accordingly, employing individual pixel points on coplanar electrodes to monitor the entire material offers a feasible means of suppression (Table 1).

**Table 1:** A comparison of common existing pressure monitoring devices.

Sensor	Hardware acquisition equipment	Sensitivity	Response time (ms)	Space resolution	Cost	Size	Ref
Self-powered triboelectric nanogenerator (TENG)	DAQ(NI-9220)	7.78 kPa^−1^	\	4*4	High	Array:1*1cm^2^ Device:8.7*11.9 cm^2^	[28]
Flexible strain sensors	DAQ	\	100	3*3	High	Array:3*3cm^2^Device:big	[46]
Super capacitive pressure sensor (SCPS)	LCR(TH2830)	0.41 kPa^−1^ (15.78–200 kPa)	200	4*4	High	Array:8.5*8.5 cm^2^ Device:36*23.5 cm^2^	[4]
Organohydrogel sensor arrays	RC signalacquisition system (01RC)	35.1 MPa^−1^ (0–20 kPa) 0.0372 MPa^−1^ (20–1500 kPa)	700	2*3	High	Device:6.8*6.8 cm^2^	[47]
Flexible pressure sensors	Self-designed	5.09 MPa^−1^ (0–25 kPa) 0.0209 kPa^−1^ (25–75 kPa)	150	8*8	Low	Array:5*5cm^2^ Device:big	[24]
Flexible pressure sensor	Self-designed	0.886 kPa^−1^ (0–180 kPa)	100	\	Low	Device:big	[23]
Wearable pressure sensors	Digital source meter (Keithley 2635B)	12.7 kPa^−1^ (0–20 kPa) 5 kPa^−1^ (20–50 kPa) 3.2 kPa^−1^ (50–100 kPa)	300	3*3	High	Device:45*21 cm^2^	[27]
Flexible pressure sensor(The Research)	Self-designed	0.628 kPa^−1^ (0–48 kPa) 0.126 kPa^−1^ (48–160 kPa)	300	4*4	Low	Array:1.8*1.8 cm^2^ Device:2.3*3cm^2^	\

On the other hand, electrode and circuit design also represent critical limiting factors for the reliability of array systems. Metallic materials are commonly used as electrodes; however, when interfaced with hydrogel-based sensors, unavoidable redox reactions may occur, leading to signal drift and long-term performance degradation. Previous studies attempted to alleviate this problem using redox buffer solutions and ion-selective membranes [39], [40], but such approaches substantially increase the contact impedance and degrade signal quality. In contrast, driving and detecting signals with alternating current can effectively suppress electrochemical reactions and enhance signal stability, making it a more practical and feasible circuit design strategy. Against this background, the development of miniaturized, multi-channel, and low-crosstalk flexible hydrogel pressure-sensing array systems holds significant promise for advancing tactile sensing applications in robotics and wearable devices.

## 2 Hardware description

### 2.1 Overview

**Fig. 1:**
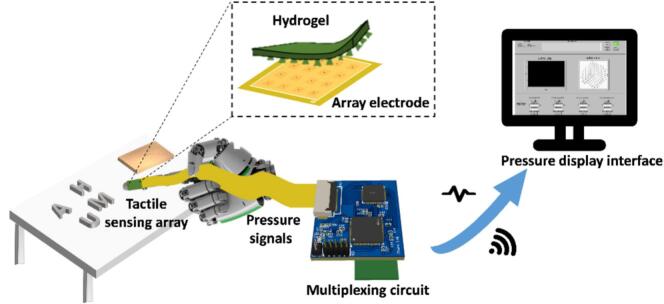
An overview of the miniature multi-channel pressure monitoring system.

The miniature multi-channel pressure monitoring system we designed consists of three main components: a flexible pressure sensing array, a wireless multi-channel acquisition system, and a pressure display interface. The flexible pressure sensing array serves as the sensing unit and is composed of hydrogel materials coupled with a coplanar square-ring electrode array. The array features inner and outer electrode layers on a substrate, offering a lightweight, compact, and simple design that effective detects external pressure variations. The multi-channel acquisition system is mainly composed of a PSoC chip, which integrates both analog and digital modules in a single chip, ensuring system portability. Through the PSoC chip, an alternating current (AC) excitation is output and applied to the functional hydrogel via the array electrodes. When external pressure alters the contact state of the hydrogel, its impedance varies, leading to synchronous changes in the detected voltage. These voltage variations are acquired by the ADC and demodulated using a digital lock-in detection technique. It then transmits the data wirelessly to the pressure display interface via the Bluetooth module. The pressure display interface visualizes the dynamic pressure changes of each pixel point in the sensing array (Fig. 1).

### 2.2 Array design

The contact electrodes were fabricated using a copper foil array on a flexible printed circuit (FPC) board. The electrode pattern was first designed using Lichuang EDA software and exported as Gerber files according to the predefined coplanar square-ring electrode structure. The FPC electrodes were then directly manufactured by a third-party professional PCB manufacturer (JLCPCB, China) using industrial standard processes. Order parameters and specifications were selected as shown in Table 2. Fabrication used a 2-layer FPC with a finished thickness of 0.11 mm, Using an adhesiveless electrolytic copper-clad laminate consisting of a 25 μm polyimide substrate and 1/3oz copper. After etching the copper array, a polyimide coverlay was applied for insulation. Finally, the exposed electrode regions underwent an Electroless Nickel Immersion Gold (ENIG) surface treatment, depositing a gold layer of approximately 1 u'' (0.0254 μm) to ensure oxidation resistance and stable contact.

**Table 2:** Selection of order parameters and specifications.

Parameters category	Specifications
Board type	Standard FPC
Finished thickness	0.11 mm
Copper type	Adhesiveless electrolytic copper foil
Coverlay	PI: 12.5 μm / AD: 15 μm
Copper thickness	1/3oz(≈12 μm)
Gold thickness	1 u'' (≈0.0254 μm)

The deposition of a 1 µm-thick gold layer on the electrode surface significantly enhanced the electrical conductivity and oxidation resistance of the electrodes. The electrode array had an overall size of 1.8 × 1.8 cm^2^ and adopted a 4 × 4 layout. A coplanar square-ring electrode structure [41] was employed in the design, consisting of independent inner electrodes and a shared outer electrode. The inner electrodes were surrounded by the outer electrode, forming a stable electric field and reducing interference. The contact electrodes formed a robust interface with the hydrogel patch, constituting a functional sensing unit capable of reliably acquiring pressure data.

### 2.3 Miniaturized multi-channel acquisition system

To achieve system miniaturization and low-power operation, a PSoC (CY8C5888LTI-LP097) chip was employed as the core device. The CY8C5888LTI-LP097 chip integrates four 8-bit current-output digital-to-analog converters (IDACs), a 12-bit analog-to-digital converter (ADC), a Cortex-M3 microcontroller unit (MCU), which significantly reduces the use of external components. Together with a Bluetooth module for wireless transmission, a voltage-regulation circuit for power management, and a multiplexer (MUX) for channel addressing, the overall system size was reduced to 2.3 cm × 3.0 cm. The excitation signal for the flexible pressure-sensing array is generated by two IDACs. One is set to “source mode” to generate positive half of the sinusoidal signal and the other is set to “sink mode” to generate negative half of the sinusoidal signal. A reference voltage of $V_{\mathrm{Ref}}$ = 1.024 V is applied to the common outer electrode of the sensor array, providing a stable bias point. The output current amplitude of each IDAC is 31.875 μA (peak) at a driving frequency of 1 kHz, which is within the safe range for skin-contact applications. The multiplexer sequentially selects the inner electrodes of each pixel in the array at a switching rate of 100 Hz, enabling time-division multiplexing of 16 channels. During each switching period, current flows through the addressed pixel to the common electrode, and the corresponding voltage response is measured. When pressure is applied to a specific pixel, its impedance changes, leading to a synchronous variation in the detected voltage. The ADC samples the signal at 100ksps with 12-bit resolution, using the reference voltage as the negative input to ensure differential measurement and accurate impedance acquisition. Fig. 4 illustrates the signal-generation and acquisition.

### 2.4 Multiplexing

The miniature multi-channel pressure monitoring system applies an alternating current and performs signal acquisition through a multiplexer. The electrode array consists of one common outer electrode and 16 inner electrodes. As shown in Fig. 5, the red lines represent the inner electrode channels, while the blue lines represent the outer electrode channel. The inner electrodes are sequentially selected via the multiplexer, with the outer electrode connected to a 1.024 V voltage source, thereby enabling the real-time acquisition of the status of each pixel in the electrode array.

### 2.5 Demodulation

The IDAC module of the PSoC generates a sinusoidal current excitation that is driven into the overlying hydrogel through an electrode array. As the local impedance of the hydrogel changes with applied pressure, the amplitude of the resulting sinusoidal voltage across the gel varies accordingly; measuring this amplitude therefore maps the pressure distribution across the micro-structure.

In this work the excitation current, set to 1 kHz, is given by expression (1).

(1) $$i\left(t\right)=31.875\cos\left(2\pi ft\right)\;\left(\mu A\right)$$

It produces a sinusoidal voltage at the electrodes as expressed in (2).

(2) $$u\left(t\right)=31.875\left|Z\right|\cos\left(2\pi ft+\theta \right)\;\left(\mu V\right)$$

where the impedance is written Z = |Z|∠θ. Because the voltage amplitude is directly proportional to |Z|, its value can be inverted from the amplitude measurement. A digital lock-in algorithm is used to extract this amplitude. Lock-in detection suppresses out-of-band interference and offers high precision; the principle—multiplying the signal by two orthogonal references, integrating, and taking the vector sum—is well documented in literature [42] and only summarized here.

An on-chip ADC samples the electrode voltage at 100 ksps (100 × the signal frequency); the DMA controller streams the data into an array to yield the digital sequence u[n]. Two reference arrays of length 100 are pre-stored:

(3) $$refI\left[n\right]=cos\left(2\pi n/100\right)\;\;\;\;0\leq n\leq 99$$

(4) $$refQ\left[n\right]=sin\left(2\pi n/100\right)\;\;\;0\leq n\leq 99$$

The lock-in multiplications and averaging are performed as

$$X_{I}=\frac{1}{M}\sum_{k=0}^{M-1}u\left[k\right]\times refI\left[k\%100\right]$$

$$X_{Q}=\frac{1}{M}\sum_{k=0}^{M-1}u\left[k\right]\times refQ\left[k\%100\right]$$

with an integration length M = 1 000, corresponding to 10 ms. Longer integration improves signal-to-noise ratio but slows response; 10 ms provides a practical compromise. The voltage amplitude is finally obtained from

$$U_{m}=2\sqrt{X_{I}^{2}+X_{Q}^{2}}$$

After lock-in detection the AC amplitude is converted to a DC value. To remove pixel-to-pixel baseline drift, the impedance change of each pixel is normalized to its initial value: |ΔZ|/|Z_0_|. The system then outputs stable, normalized impedance data for further analysis.

### 2.6 Pressure display interface design

The upper-computer pressure display interface is implemented using LabVIEW. The pressure display interface consists of three functional modules: a device connection module, a data processing and visualization module, and a data storage module. The device connection module transfers the collected data from the multiplexer circuit to the display interface via a Bluetooth module and a virtual serial port. The data processing and visualization module calculates the relative changes of each array pixel by comparing the impedance variations with the initial impedance values, and presents the results intuitively in the form of intensity maps. The data storage module enables the saving and exporting of required data, facilitating subsequent analysis and processing.

## 3 Design files summary

Table 3 lists the locations of all hardware and software design files required to reproduce and operate the device. All files are available in the repository.

**Table 3:** Design files of miniature multi-channel pressure monitoring system.

Design file name	File type	Open source license	Location of the file
Array Electrode	PCB Gerber	CC BY 4.0	https://data.mendeley.com/datasets/6n9t58g2v3 “Hardware_PCB file”
PCB of acquisition circuit	PCB Gerber	CC BY 4.0	https://data.mendeley.com/datasets/6n9t58g2v3”Hardware_PCB file”
Pressure display interface	VI	CC BY 4.0	https://data.mendeley.com/datasets/6n9t58g2v3”LabVIEW_interface file”
PSoC software	PSoC Creator Workspace	CC BY 4.0	https://data.mendeley.com/datasets/6n9t58g2v3”PSoC_software file”
BLEComManager	Executable	CC BY 4.0	https://data.mendeley.com/datasets/6n9t58g2v3”BLEComManager file”
Scheme of current source in PSoC editor	Figure PNG	CC BY 4.0	Included in the paper (Fig. 4)
FPC array	Figure PNG	CC BY 4.0	Included in the paper (Fig. 2)
Circuit board	Figure PNG	CC BY 4.0	Included in the paper (Fig. 3)
Acquisition system	Figure PNG	CC BY 4.0	Included in the paper (Fig. 7)
Multiplexing	Figure PNG	CC BY 4.0	Included in the paper (Fig. 4)
Pressure display Interface configuration	Figure PNG	CC BY 4.0	Included in the paper (Fig. 6)
PSoC_Creat operation	Figure PNG	CC BY 4.0	https://data.mendeley.com/datasets/6n9t58g2v3”PSoC_software file”
BLEComManager operation	Figure PNG	CC BY 4.0	https://data.mendeley.com/datasets/6n9t58g2v3”Operation_instructions file”
Pressure display interface operation	Figure PNG	CC BY 4.0	https://data.mendeley.com/datasets/6n9t58g2v3”Operation_instructions file”

## 4 Bill of materials summary

The list of materials used in the design of the miniature multi-channel tactile sensing and monitoring system is presented in Table 4.

**Table 4:** Bill of materials of the miniature multi-channel pressure monitoring system.

Designator	Component	Number	Cost per unit − currency (RMB)	Total cost − Currency(RMB)	Source of materials	Material type
PSoC 5LP (CY8C5888LTI-LP097)	Programmable SoC, combining high-precision analog and digital peripherals	1	134.83	134.83	taobao.com	Electronic
Multiplexing	TS3L4892RHHR	1	8.46	8.46	szlcsc.com	Electronic
Bluetooth	BLE-SER-A-ANT	1	16.65	16.65	szlcsc.com	Electronic
LDO	ADP160ACBZ-3.3-R7	1	6.02	6.02	szlcsc.com	Electronic
Battery lithium 5 V	Battery	1	6.9	6.9	taobao.com	Other
Total				172.86		

## 5 Build instructions

**Fig. 2:**
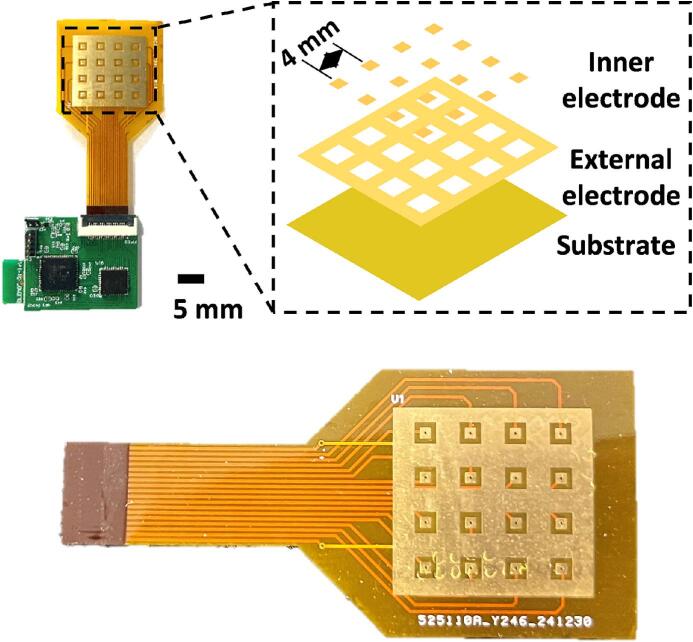
Copper foil array electrode.

The electrode array was fabricated using the PCB design provided in the “Gerber_ConGND_FPC” file. JLCPCB (Guangdong, China) can produce this circuit board, as shown in Fig. 2. It is necessary to select FPC and gold plating processes for production.

**Fig. 3:**
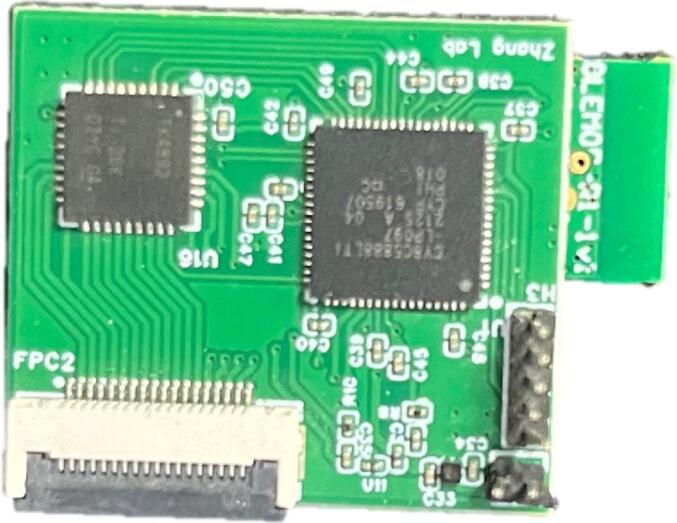
Wireless multi-channel acquisition circuit board.

The miniature multi-channel acquisition system can order the PCB on JLCPCB or similar platforms using the “Gerber_Hardware_PCB” file. The soldering can be completed by placing an SMT assembly order at JLCPCB, which will deliver the fully assembled board, as shown in Fig. 3. Alternatively, electronic components can be purchased from SZLCSC, solder paste can be applied to the pads, and then soldering can be carried out using a reflow soldering machine.

**Fig. 4:**
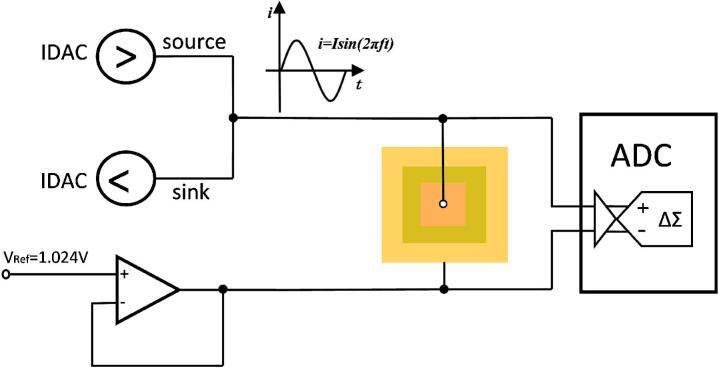
Sinusoidal signal generator based on PSOC IDAC and ADC signal acquisition.

**Fig. 5:**
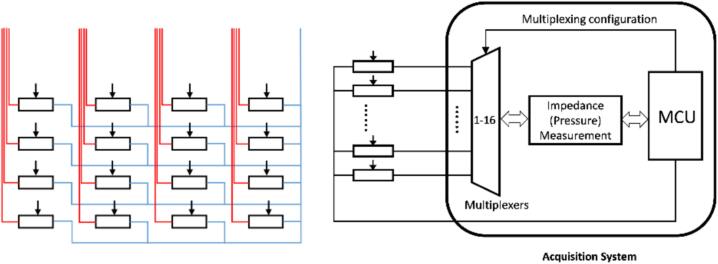
Multiplexing scheme.

The assembled miniature multi-channel acquisition board is then programmed. The software for this study was developed using PSoC Creator 4.4, which can be downloaded from the Cypress website: https://www.cypress.com/products/psoc-creator-integrated-design-environment-ide. The acquisition board is connected to a computer via a USB-to-TTL interface, and the “current_output” project file in the “PSoC_software” folder is opened. The operation steps can be found in the “PSoC_software” folder under the “PSoC_Creat” diagram. In the Pins tab, the device model CY8C5888LTI-LP097 is selected, the pin connections are verified, and after configuration, the firmware can be programmed by selecting the Debug option in the software. Before programming, ensure that the correct device model is selected in the Device Selector and verify that all pin configurations in the Pins tab match the hardware connections to ensure a successful firmware download.

The FPC connector on the miniature multi-channel acquisition system is then connected to the array-structured board. As shown in Fig. 7, the assembled device is obtained by inserting the gold finger portion of the array board into the FPC connector socket on the acquisition board. Finally, battery power is supplied by connecting to the VCC and GND pins.

**Fig. 6:**
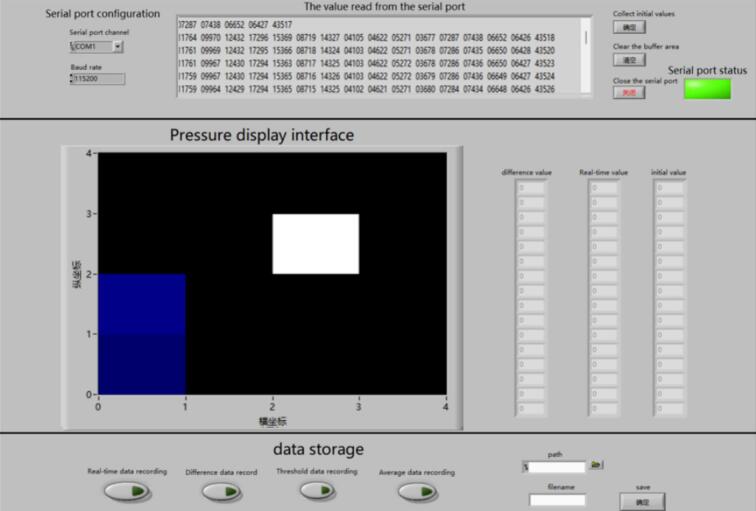
Pressure display interface.

**Fig. 7:**
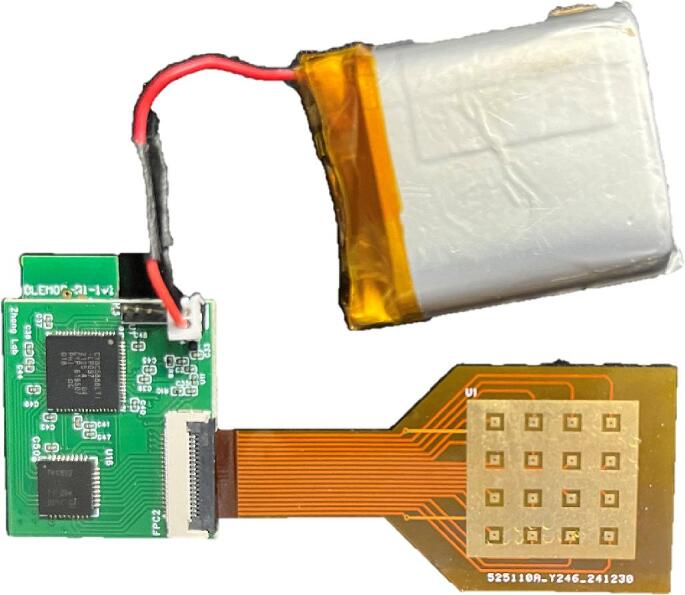
Wireless multi-channel acquisition system.

## 6 Operation instructions

**Fig. 8:**
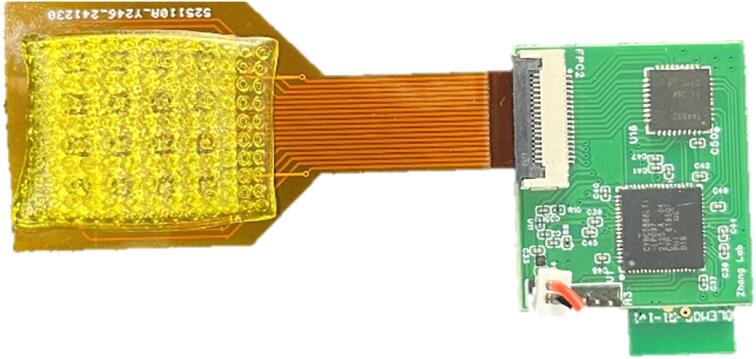
System diagram for placing the hydrogel.

The hydrogel is first carefully positioned onto the thin, flexible FPC board. Then, the multi-channel acquisition system module is connected to the electrodes, and a 5 V power supply is applied to the circuit to initiate operation. During connection, ensure the correct polarity of the battery; reverse connection or short-circuiting must be avoided to prevent circuit damage or lithium battery failure. Additionally, care should be taken when handling the FPC board to avoid scratching or mechanical damage. While the hydrogel material exhibits excellent biocompatibility, safety precautions are still required in specific applications to ensure aseptic conditions and prevent adverse reactions. The acquired data are transmitted via Bluetooth, as shown in Fig. 8.

Enable Bluetooth on the host computer and use BLEComManager to open the Bluetooth virtual serial port, allowing the computer to read data from the virtual Bluetooth COM port. BLEComManager can be installed by opening the BLEComManager folder located in the Design Files directory. The operation examples diagram are contained in the “Operation_instructions” file.

The upper-computer interface is implemented using LabVIEW. LabVIEW should be installed first, and the VISA module for serial communication should be searched for and installed via the NI Package Manager. The software used in this research was developed using LabVIEW. This software can be downloaded from the NI website: https://www.ni.com/zh-cn/support/downloads/software-products/download.labview.html. Installation of LabVIEW and VISA is described in the help information on the NI website.

As shown in Fig. 6, After launching LabVIEW, open the Pressure display interface located in the “LabVIEW_interface” folder. The operation examples diagram are contained in the “Operation_instructions” file. In the “Serial port channel” select the Bluetooth virtual serial port number, and set the “Baud rate” to 115200. After the correct selection and configuration, start running the program. When the program is running, baseline data are automatically acquired; baseline initialization and acquisition can also be repeated using “Collect initial values”. The value of each array element can be monitored through “The value read from the serial port”, and the displayed values can be cleared using “Clear the buffer area”. The real-time pressure distribution at each array element can be observed in the “Pressure display interface”, where different pressure levels are represented by different colors, as shown in Fig. 6. For data storage, select the data to be saved, specify the file save path in “Path”, and define the file name in “Filename”. Click “Save” to store the required data; files must be saved while the program is running. To terminate the program, click “Close the serial port” to end operation.

## 7 Validation and characterization

In this study, a diagnostic–therapeutic integrated hydrogel patch (PAMS) was employed as the core material for the flexible pressure sensor [43]. The patch exhibits excellent biocompatibility and bioadhesiveness, enabling it to establish a stable and tight mechanical–electrical coupling with the contact electrodes, which significantly enhances its performance in human–machine interface applications. Moreover, it demonstrates outstanding mechanical–electrical properties, with high deformability and sensitivity. The key features are summarized in Table 5.

**Table 5:** PAMS characteristics.

Parameter	Value
Conducting material	MXene
Sensor thickness	2.5 mm
Electrical conductivity	4.03*10^−4^ Scm^−1^
Sensitivity	GF = 4.73
Attachment strength	11.26 kPa
Maximum deformation	460%

### 7.1 Crosstalk and corrosion

**Fig. 9:**
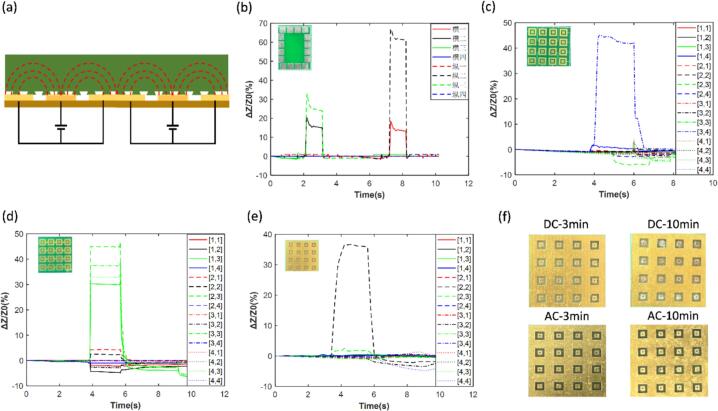
Design of anti-interference array. (a) Electric field distribution map of the pressure sensing array. (b) Measurement of the pressing of 4 horizontal and 4 vertical channels of the surrounding reference electrodes. (c) Measurement of the pressing of 16 channels of a single array pixel point. (d) Measurement of the pressing of 16 channels of the row and column structure electrodes. (e) Measurement of the pressing of 16 channels of the independent current output and coplanar voltage structure electrodes. (f) The corrosion conditions of direct current (upper picture) and alternating current (lower picture) under 3 min (left picture) and 10 min (right picture) of application.

When using array structures to measure pressure distribution, various interfering factors can introduce deviations into the measurement data. To mitigate these issues, we employed a coplanar square-loop electrode array, which reduces inter-channel crosstalk and provides a stable electric field to minimize external noise interference [41], [44], [45]. To evaluate different electrode configurations, we designed and analysed four electrode structures. The first adopts an opposing electrode design, commonly used in electrical impedance tomography, this design applies input and output signals to opposing electrodes. As shown in Fig. 9b, it can detect central compression changes, but measurements near the edges suffer from contact resistance interference. In addition, electrode spacing increases array size, making this design unsuitable for miniaturization and high-precision applications. Therefore, we have designed an array electrode with a square ring structure, where the changes in the contact electrodes play a dominant role. The outer ring array electrode can provide a stable electric field to reduce the interference from external noise. The second type adopts a separate array pixel point measurement design. Each pixel is equipped with separate inner (input) and outer (output) electrodes, with spacing between adjacent pixels to isolate current pathways. This reduces mechanical and electrical crosstalk, allowing clear localization of pressing positions (Fig. 9c). However, the large number of connections substantially increases circuit area and complexity. The third design employs a row-column electrode configuration. Here, pixels in the same row share input electrodes, while those in the same column share output electrodes. This reduces the number of channels but introduces electrical interference along shared pathways, leading to unintended voltage variations, as shown in Fig. 9d. The fourth design utilizes Independent input with common output. This design employs 16 independent input channels with a common planar output electrode. As illustrated in Fig. 9e, it enables accurate pressure localization without significant crosstalk, while balancing circuit complexity and channel count. The use of an independent array can effectively reduce electrical crosstalk. It is possible to further reduce the number of channels by connecting the outer electrodes, or to increase the number of channels to make the array more refined.

When hydrogels are in contact with metal electrodes, an electrochemical galvanic effect may occur. Due to the persistent potential difference during operation, redox reactions take place at the electrode surface, leading to anode corrosion. As illustrated in Fig. 9f, under a 1.024 V direct current (DC) excitation for 3 min and 10 min, anode corrosion is observed, which increase electrode-related measurement drift and may ultimately result in electrode failure, thereby compromising the accuracy of the data. To address this issue, we employed a 1 kHz alternating current signal as the excitation source. After the same 3 and 10 min intervals, almost no corrosion is observed. The periodic reversal of the AC waveform effectively inhibits the galvanic effect and reduces signal interference caused by electrode corrosion. In addition, we shaped the cathode as a continuous, large-area electrode; the increased surface further lowers the current density, slows corrosion, and improves measurement stability.

### 7.2 Pressure sensor characteristics

**Fig. 10:**
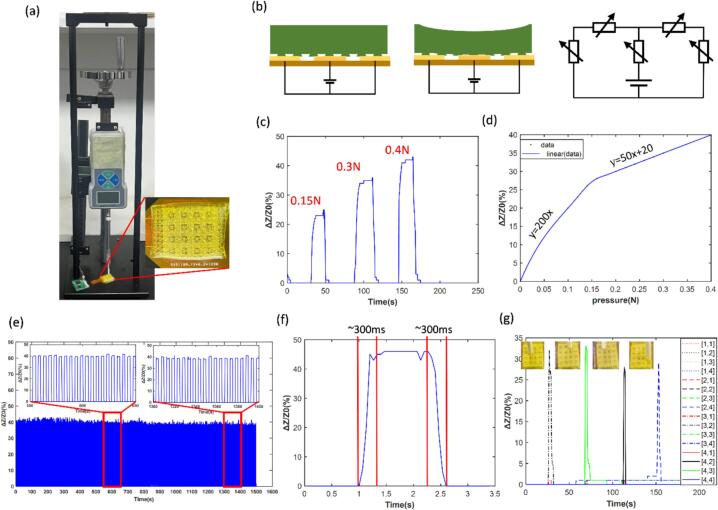
Sensing performance of the pressure sensor. (a) Experimental setup and photograph of the pressure sensor. (b) Working mechanism of the pressure sensor. (c) Changes in impedance values under different pressures. (d) Curves of pressure and impedance values. (e) Durability and stability of pressure signal measurement. (f) Response/recovery time of the pressure sensor. (g) Changes in 16 channels when different pixels are pressed.

We experimentally validated the pressure-sensing characteristics of the miniaturized multi-channel pressure monitoring system. The experiment employed a push–pull force gauge combined with an insulated rod with a radius of 1 mm for the pressing test, as shown in Fig. 10a. The pressure sensing mechanism of the hydrogel array is shown in Fig. 10b. Under external pressure, the contact area between the hydrogel and the array electrodes increases, thereby leading to a gradual reduction in contact resistance. As shown in Fig. 10c, we conducted tests using different forces for pressing measurements. The relative impedance changes collected under three pressing forces of 0.15 N, 0.3 N, and 0.4 N were examined. The relative impedance change values can clearly reflect the variations of different magnitudes of forces. It can be observed that each pixel point of the array has a high sensitivity and can perceive small changes in force. As shown in Fig. 10d, the curve representing the relative change in impedance as the pressure increases from 0 N is measured. When the applied pressure is below 0.15 N, the relative change in impedance is relatively significant and has high sensitivity, indicating sensitivity of 0.628 kPa^−1^ (0–48 kPa). When the applied force is greater than 0.15 N, the relative change in impedance is relatively stable with a reduced sensitivity of 0.126 kPa^−1^ (48–160 kPa), but the measurement range of the entire system is relatively small, ranging from 0 to 0.5 N (0–160 kPa). As shown in Fig. 10e, we performed 300 consecutive compression cycles on a single array pixel, with a cycle period of 5 s, during which pressure was repeatedly applied and released while the response was recorded. The applied pressure was 0.4 N. No performance degradation was observed as the number of compressions increased, meeting the stability requirements for repeated operation. This pressure-sensitive array demonstrates good repeatability and reliability. As shown in Fig. 10f, when pressure is applied to the array, the response time of the system to the rising and falling edges of the pressure impedance curve is approximately 300 ms. This delay is influenced by the lock-in integration time and the intrinsic hydrogel material properties. Experiments have proved that this system can achieve high-precision and fast response for pressure perception.

As shown in Fig. 10g, we conducted an experimental verification of the spatial consistency of the array. Four different pixel positions ([3,2], [4,3], [4,2], [2,4]) were randomly selected and subjected to localized pressing. The results show that when a specific pixel is pressed, only the corresponding pixel exhibits a relative impedance change, which is rapidly reflected during the pressing process. Moreover, under the same applied force, the response magnitudes at different pixel positions remain largely consistent. The results demonstrate that the pressure sensor array not only achieves high-precision recognition and localization of pressure positions but also ensures reliable spatial consistency.

### 7.3 Pressure recognition in the pressing mode

**Fig. 11:**
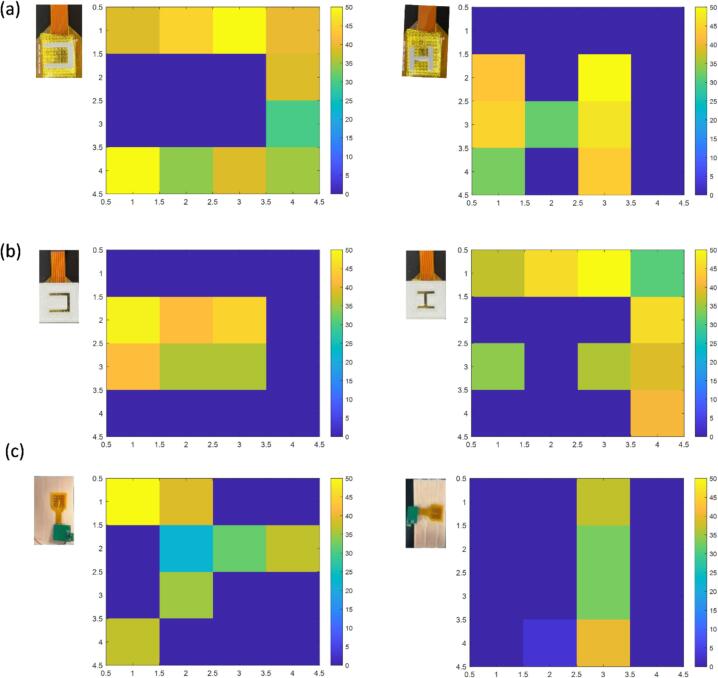
The pressure measurement and display interface of the tactile pressure sensor. (a) When pressing the uppercase letters “U” and “H”, the display graphics of the pressure display interface. (b) When pressing the lowercase letters “U” and “H”, the display graphics of the pressure display interface. (c) The images displayed on the pressure display interface when touching the Y-shaped blood vessel and the straight-shaped blood vessel.

This study further evaluated the capability of the miniature multiplexed pressure detection system in pressure shape recognition. By applying objects with different shapes onto the sensor array and analysing the resulting relative impedance changes, the system was able to reconstruct and identify the external shapes of the pressed objects. To validate this, 3D-printed models of the letters “H” and “U” in both embossed and engraved forms were fabricated as test samples. The embossed model had a letter size of 13 mm × 15 mm with a contour width of 2 mm, while the engraved model had a letter size of 20 mm × 20 mm with a contour width of 3 mm. As shown in Fig. 11a and b, the array accurately distinguished the shapes of the 3D-printed letters, and the recognition results were clearly reflected in the obtained intensity spectra. These findings demonstrate that the proposed multi-channel monitoring system exhibits excellent pressure shape recognition capability, enabling accurate identification of pressed objects.

For the touch-press measurement of the micro multi-channel pressure detection system, as shown in Fig. 11c, by placing the pressure sensor array on the simulated skin and using fingers to press the array, the vascular outline can be displayed through the pressing measurement. We conducted compression measurements on both straight vessels and Y-shaped bifurcated vessels. The results showed that the array was capable of accurately identifying and presenting the morphological characteristics of different vascular structures, and achieving the recognition of the pressure on the compressed part through touch and compression. The experimental results show that this miniature multi-channel pressure detection system can monitor the pressure conditions at the skin detection area.

## CRediT authorship contribution statement

**Zhuwen Xu:** Writing – review & editing, Writing – original draft, Visualization, Validation, Supervision, Software, Resources, Project administration, Methodology, Investigation, Formal analysis, Data curation, Conceptualization. **Yin Zhang:** Validation, Investigation, Formal analysis, Data curation. **Zeyu Liu:** Writing – review & editing, Supervision, Investigation, Formal analysis, Data curation. **Xingqi Lu:** Resources, Methodology, Formal analysis. **Runhuai Yang:** Supervision, Project administration, Formal analysis, Conceptualization. **Shengzhao Zhang:** Writing – review & editing, Validation, Supervision, Resources, Project administration, Methodology, Investigation, Funding acquisition, Data curation.

## Declaration of competing interest

The authors declare that they have no known competing financial interests or personal relationships that could have appeared to influence the work reported in this paper.
